# Recognition and Management of Antipsychotic-Induced Parkinsonism in Older Adults: A Narrative Review

**DOI:** 10.3390/medicines8060024

**Published:** 2021-05-26

**Authors:** Sharadha Wisidagama, Abiram Selladurai, Peter Wu, Marco Isetta, Jordi Serra-Mestres

**Affiliations:** 1 Departments of Psychiatry, Central and North West London NHS Foundation Trust, London NW1 3AX, UK; sharadha.wisidagama@nhs.net (S.W.); aselladurai@nhs.net (A.S.); peter.wu@nhs.net (P.W.); 2 Knowledge and Library Services, Central and North West London NHS Foundation Trust, London NW1 3AX, UK; marco.isetta@nhs.net; 3 Old Age Psychiatry, Central and North West London NHS Foundation Trust, Uxbridge UB8 3NN, UK

**Keywords:** parkinsonism, antipsychotic drugs, older adults

## Abstract

Background: Parkinsonism is a common side-effect of antipsychotic drugs especially in older adults, who also present with a higher frequency of neurodegenerative disorders like Idiopathic Parkinson’s disease (IPD). Distinguishing between antipsychotic-induced parkinsonism (AIP) and IPD is challenging due to clinical similarities. Up to 20% of older adults may suffer from persisting parkinsonism months after discontinuation of antipsychotics, suggesting underlying neurodegeneration. A review of the literature on AIP in older adults is presented, focusing on epidemiology, clinical aspects, and management. Methods: A literature search was undertaken on EMBASE, MEDLINE and PsycINFO, for articles on parkinsonism induced by antipsychotic drugs or other dopamine 2 receptor antagonists in subjects aged 65 or older. Results: AIP in older adults is the second most common cause of parkinsonism after IPD. Older age, female gender, exposure to high-potency first generation antipsychotics, and antipsychotic dosage are the main risk factors. The clinical presentation of AIP resembles that of IPD, but is more symmetrical, affects upper limbs more, and tends to have associated motor phenomena such as orofacial dyskinesias and akathisia. Presence of olfactory dysfunction in AIP suggests neurodegeneration. Imaging of striatal dopamine transporters is widely used in IPD diagnosis and could help to distinguish it from AIP. There is little evidence base for recommending pharmacological interventions for AIP, the best options being dose-reduction/withdrawal, or switching to a second-generation drug. Conclusions: AIP is a common occurrence in older adults and it is possible to differentiate it from IPD. Further research is needed into its pathophysiology and on its treatment.

## 1. Introduction

Extrapyramidal symptoms are well-known side-effects of dopamine 2 receptor (D2R) antagonist drugs, which act on D2Rs in the striatum and mesocortex [1], and comprise a range of acute and tardive motor phenomena, from rigidity, tremor and bradykinesia, to dystonia, dyskinesias, and akathisia. Drug-induced parkinsonism (DIP) refers to a conglomerate of signs that are very similar to, and at times difficult to distinguish from, Idiopathic Parkinson’s disease (IPD), and that usually appears and evolves over a period of a few weeks after drug initiation or increase [2]. DIP was first reported following the introduction of antipsychotic drugs (also known as neuroleptics) (APs) as treatment for psychoses in the 1950s, and is more commonly seen with high-potency first generation antipsychotics (FGAs) such as haloperidol [3]. Most cases of DIP are associated with the use of FGAs (especially high-potency ones), and less frequently with second generation antipsychotics (SGAs). However, DIP has also been associated with other families of compounds such as anti-nausea drugs (e.g., substituted benzamides, etc.), calcium channel blockers, sodium valproate, and lithium. Although recovery from DIP after withdrawal of the offending drug may take weeks or months, complete recovery is not certain, and around 20% of patients may continue to experience symptoms thereafter [4]. The risk of developing DIP has been reported to increase with age [5]. In addition, older people are also at a higher risk of emerging neurodegeneration which could compound the differential diagnosis of parkinsonism. In this sense, DIP might sometimes represent the “unmasking” of subclinical nigrostriatal dysfunction secondary to IPD or another α-synucleinopathy [6].

A review of the literature was undertaken in order to ascertain the epidemiology of DIP in older adults (aged 65 or older) caused by APs (antipsychotic-induced parkinsonism—AIP) as well as D2R antagonists; and establish its clinical characteristics, differential diagnosis, management, and prognosis. 

## 2. Methods

A narrative review of the literature reporting on parkinsonism secondary to antipsychotic drugs and other D2R antagonists (such as benzamides) in older adults (aged 65 or older). The search was limited to English articles, or in other languages if English abstracts were available. Editorials, conference papers, letters to the editor and single case reports were excluded.

### Database Search Methodology

Systematic literature searches were performed in the three core healthcare databases: EMBASE and MEDLINE (OVID interface) on 29 December 2020; and PsycINFO (via the NICE HDAS interface) on 30 December 2020. All searches were repeated on 25 February 2021 to capture any recent publications. In order to maximise sensitivity, the search strategies relied on blended subject headings and keyword (free text) approaches—with search language adapted to each database’s own syntactical choices. 

On MEDLINE, applicable MESH headings (PARKINSON DISEASE, SECONDARY/ and exp ANTIPSYCHOTIC AGENTS/) were supplemented by natural language (neuroleptic-induced parkinsonism.ti,ab. or antipsychotic-induced parkinsonism.ti,ab. or drug-induced parkinsonism.ti,ab.). Similarly, the above free-text strings were added to PARKINSONISM/ and (exp NEUROLEPTIC AGENT/ or exp DRUG INDUCED DISEASE/ in EMBASE; and combined with PARKINSONISM/ and exp “SIDE EFFECTS (DRUG)”/ in PsycINFO. Age limits (65 years and over) and validated search filters for the aged 65 and older—as reviewed by the InterTASC Information Specialists’ Sub-Group (ISSG) were applied across all three searches” (e.g., OVID Medline (aged/ or “aged, 80 and over”/ or frail elderly/) OR (elderly or “over 65” or “over 80” or “65 year*” or “85 year*”).ti,ab).

## 3. Results with Discussion

**Figure 1:**
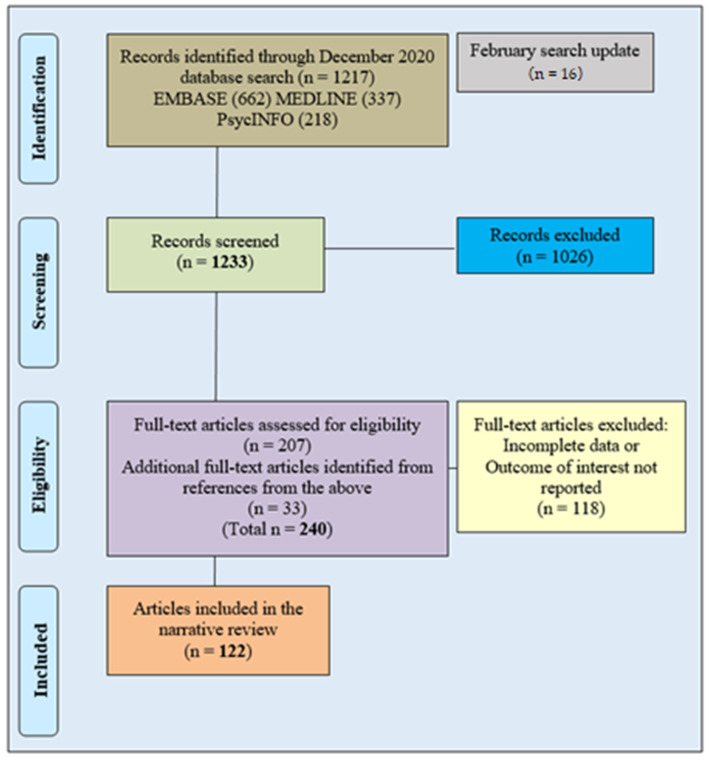
Article search and selection flowchart.

All the retrieved findings (initial searches: EMBASE 662; MEDLINE 337; PsycINFO 218; updated search: 16 for the three databases) were reviewed separately by the authors on agreed inclusion criteria. Individual shortlists were cross-checked and discussed jointly, resulting in a final list of 207 articles for sourcing. 22 selected items could not be obtained as full-text. Additional papers (33) were sourced after revising the selected articles’ reference lists. A total of 122 articles were included in the final review. See Figure 1 for a flowchart illustrating search and selection method.

### 3.1. Epidemiology and Risk Factors

Prevalence and incidence rates of DIP vary depending on the ascertainment method and population studied [7], but it has been suggested that globally over 50% of over 60 years old subjects on long-term therapy with APs develop AIP [8]. For example, among 906 incident cases of parkinsonism in a US community study from 1976 to 2005, 108 persons had DIP (11.9%) [9]. This study found the incidence of DIP to have increased in older age and in women, but to have decreased overall in that period due to changes in the prescription of APs [9]. Other studies have, however, provided evidence for an overall increase in the annual prevalence of DIP; for example, from 4.09/100,000 in 2009 to 7.02/100,000 in 2015 in a South Korean study [10], which was mainly caused by the use of benzamide derivatives for gastrokinetic indications. See Table 1a,b for the relevant epidemiological studies.

Most published studies in community or general population settings have reported DIP to be the second (or less frequently third) most common cause of parkinsonism after IPD, especially in the older population, ranging from 2% to 76% of all cases of parkinsonism [5,9,10,11,12,13,14,15,16,17,18,19,20,21,22,23,24,25]. However, when a large database of over 20000 cases of drug-induced side-effects over a 17-year period was examined in one French study, the rate of DIP was found to be of 0.7% [26]. In a recent pharmacovigilance study using the World Health Organisation (WHO) database between 2000 and 2017, DIP was reported in 0.05% across age groups, with the highest rates in the over 45 age group [27]. In another pharmacovigilance study involving a database of 245,958 patients treated with APs between 2001 and 2016, there were 200 adverse reactions of severe parkinsonism reported, giving an overall rate of 0.08% [3].

Population studies of prevalence have reported rates of DIP of 9.78/100,000 [28], 4.09–7.02/100,000/year in a period of 6 years [10], 21.7/100,000 [25], 37/100,000 [29] and 0.5/100 [30]. Another study in Sicily over a 4-year period showed a prevalence of 32.7/100,000 [13]. In respect of the annual average incidence of DIP, a population study in Olmsted County, US, reported 3.3/100,000/year [9,23]. Incidence was higher in older age groups, ranging from 9.4/100,000 in the 60–69 age bracket to 29.3/100,000 in the 80–99 age bracket [9]. A study in Korea found an incidence rate of 8.69/100,000 over a three-year period [28], with incidence increasing with age up until the age bracket 75–79y (higher in women than men), and decreasing slightly from age 80. Finally, in a study from Taiwan using data from the National Health Insurance Research Database over the period 2000–2012, the incidence of DIP was found to be of 762.2/100,000 in patients who had taken sulpiride (a highly selective D2R antagonist) for the management of gastro-oesophageal reflux disorder, compared to those who were not treated with this drug [31]. When looking at age groups, the Olmsted study showed an increasing incidence of DIP from age 60, with the highest incidence in women in the age group 80–89 [14]: 31.8/100,000/year in men and 60.8/100,000 in women. In a Swiss study, the crude annual incidence of DIP was 2.5/100,000 [25]. An incident rate of 76% was reported in a study of AIP in Alzheimer’s disease patients [32].

Most studies report that increasing age (for both sexes) and female gender are more frequently associated with incidence and prevalence of DIP [8,9,10,14,16,17,23,25,26,27,28,31,33]. However, the vast majority of these studies include all drug groups associated with DIP, hence it is difficult to ascertain the precise prevalence and incidence of AIP.

The frequency of AIP in nursing home settings is thought to be under-reported. In a small study, 23 out of 27 (85.1%) residents on APs showed parkinsonism compared to 43 out of 73 of those not on APs [34], whilst in another study in New York City, the rate of drug-induced tremor in 397 nursing home residents was found to be 3%, although the involved medications were not APs but sodium valproate, lithium, and calcium channel blockers [35]. In the same study, the overall prevalence of DIP was 0.5% [35]. A similar rate of 3% DIP in older adults in care settings was reported in another study [36]. 

#### 3.1.1. Drugs

The majority of epidemiological studies do not distinguish, in their results, between drugs causing DIP, thus making it difficult to ascertain an accurate rate of AIP. However, a study reporting on a cohort of 1528 patients with parkinsonism where DIP accounted for 7.9% of all cases concluded that DIP was caused by APs in 53% of cases [20]. In another large study using a pharmacovigilance database, DIP was associated with D2R antagonists in 50% of cases, the rest being associated with other drugs [26]. 

In relation to types of APs, a recent pharmacovigilance study (involving a database of 245958 patients treated with APs between 2001 and 2016) found that low-potency FGAs were significantly less often implicated in the causation of AIP (0.02%) than SGAs (0.07%), and high-potency FGAs (0.16%). Among the SGAs, amisulpride and risperidone ranked highest. Phenothiazines showed significantly lower rates of severe parkinsonism (0.02%) than butyrophenones (0.11%) and thioxanthenes (0.12%) [3]. It has also been reported that the risk of AIP with high-dose SGAs does not seem to differ substantially to that from FGAs [37], and that the profile of symptoms SGAs induce is similar to that of FGAs [38]. Sulpiride, risperidone, olanzapine and haloperidol are the most frequently reported APs in AIP [27,28,39]; whilst levosulpiride, metoclopramide, clebopride and itopride, are the most frequently reported gastrokinetic drugs associated with DIP [39,40]. Data from adverse event reporting systems also points towards an increase in DIP over time, at the expense of gastrokinetic drugs and APs [41]. The incidence rates for FGA related AIP in older adults varies from 30% to 60% [42]. The incident rates for SGA related AIP is lower. Studies investigating quetiapine reported an incident rate of 6% [43], and olanzapine reported rates of 5–14% [44]. The incidence of risperidone induced AIP increased from 6.7% for 0.5 mg/day to 21.2% for 2 mg/day [45]. In a retrospective cohort study investigating dementia patients, risperidone and olanzapine taken at 10 mg/day and 2 mg/day respectively, showed a similar risk of parkinsonism compared to FGAs [37]. Considering the effects of different drug groups on the emergence of DIP, it has been suggested that there may be a bimodal pattern of symptom occurrence, with DIP caused by D2R antagonists such as APs and gastrokinetic drugs appearing within 0 to 6 months of treatment commencement; and DIP secondary to calcium channel blockers arising after 9–12 months of treatment [46,47]. This suggests that AIP arises soon after treatment is started. See Table 2 for the different AP groups, and Table 3 for studies on drugs associated with DIP and their respective frequencies.

#### 3.1.2. Risk Factors

In addition to age over 60 and female gender, a number of studies have suggested that other patient and drug-related factors can be associated with an increased risk of developing AIP (Table 4). Patient-related factors are organic brain damage, learning disability, dementia, IPD, hypertension, organic personality disorder, schizophrenia, mania, depression or anxiety, HIV infection, non-European ancestry, and presence of HLA-B44 [3,31,33,47,48,49]; whilst drug-related causes are high potency APs, higher dosage of APs, and long-term exposure to APs [3,33]. The diagnosis of DIP has also been reported to triple the lifetime risk of developing IPD [50,51], whilst the relative risk of developing IPD was found in one study to be of 24.3 (CI 95%) [52].

### 3.2. Pathophysiology

It has been long established that APs block cerebral D2Rs in the brain, especially in the striatum and mesocortex, causing a state equivalent to dopamine deficiency which is responsible for the parkinsonism [1,47]. This is the mode of action of high-potency FGAs, due to their high antagonistic affinity for D2Rs in the striatum (e.g., the butyrophenone haloperidol). However, the pathophysiology of AIP is yet to be fully understood [33]. Dopamine blockade by itself does not account for AIP completely, as symptoms of parkinsonism may last for weeks to months, whereas its effects on psychotic symptoms only last for several hours [8,53]. It is not well understood why AIP occurs after days to weeks of being exposed to an AP, whilst D2R blockade occurs minutes after exposure [47]. 

It has been proposed that the rate at which APs dissociate from the D2R (dissociation constant), may predict their likelihood to cause parkinsonism [54,55,56]; hence APs producing a transient D2 blockade (fast dissociation), such as clozapine and quetiapine, would allow the physiological stimulation of the D2R by endogenous dopamine [47,55,56] reducing the effects on the motor system whilst maintaining antipsychotic action [56]. This principle of transient occupation of D2Rs has been well demonstrated in the case of clozapine [56], and has been used to develop extended AP dosing (e.g., every other day) for other APs with slow dissociation with safe and effective clinical results [56,57]. It has also been suggested that D2R and serotonin 5-HT2A receptor blockade may reduce the risk of AIP [47]. Many SGAs block both types of receptor, and this dual effect may account for the lower reported risk of AIP associated with these agents [47,58]. Blockade of both dopaminergic and cholinergic transmission may also decrease the risk of AIP [47], as high potency FGAs such as haloperidol have little anticholinergic activity and are more likely to cause AIP compared to lower potency first generation APs (such as chlorpromazine) that have significant anticholinergic activity and less propensity to cause AIP [47]. However, studies on clozapine have shown that the effects on these other neurotransmitter systems are not the explanation for its lack of propensity to cause parkinsonism, whilst fast dissociation is [56].

Further explanations for the observation that D2 blockade by itself does not account for AIP completely come from studies of gene expression after AP intake, which have shown different patterns of gene expression in patients with extrapyramidal side-effects (EPS), including parkinsonism [59,60,61,62]. In particular, pathways involving mTOR and NF-kB kinases have been described as playing a key role in AP-induced EPS including AIP [59,60]. Further recent studies examining the effects of single nucleotide polymorphisms (SNPs) affecting transcription factor binding sites (TFBS) in response to treatment with APs, showed that two SNPs in two genes, LSMAP and ABL1, were significantly associated with AP-induced EPS [62], as did the mTOR pathway-related genes AKT1 and RPTOR [61].

Drugs that block D2Rs may also have a direct neurotoxic effect on dopaminergic neurons, and this may also explain why some patients continue to experience parkinsonism many months following discontinuation of the offending drug (around 20% of patients) [8,33,63]. This neurotoxic hypothesis is further supported by the finding of an increased long-term risk of developing IPD in older patients with a past history of exposure to APs [50], and by the findings in animal models of neuronal death with exposure to APs, likely via inhibition of the mitochondrial respiratory chain, increased D2R turnover, and free radical production [8,63]. It has also been proposed that parkinsonism occurring after a short exposure to APs may be related to an underlying dysfunction in the nigrostriatal dopaminergic pathway as ascertained through dopamine transporter (DaT) Positron Emission Tomography (PET) scanning [64]. This is highly relevant in older patients [8].

An MRI study found that patients with DIP presented with disruption of the white matter microstructure (measured by fractional anisotropy and mean diffusivity) compared to control subjects but not to IPD patients; and that these abnormalities were correlated with clinical signs of parkinsonism and presence of cognitive dysfunction, independently from exposure time to the offending drug and duration of DIP [65]. White matter abnormalities in frontal and parietal areas were more closely associated with severity of parkinsonism, and it was hypothesized that such abnormalities may disrupt the connections of functionally important basal ganglia-motor/sensory cortical circuits relevant for motor control [65]. Although causality could not be inferred from this study due to its cross-sectional design, it was suggested that microstructural alterations in the white matter may represent a pre-existing pathology of DIP, and therefore act as a risk factor for DIP, rather than being a secondary phenomenon of dopamine depleting effects [65]. In addition, a recent study reported that in patients with DIP there was decreased functional connectivity (FC) between the sensorimotor network and widespread cortical regions compared to control subjects [66]. The study also found that FC between the sensorimotor network and the prefrontal cortex correlated negatively with parkinsonian motor severity [66].

### 3.3. Clinical Characteristics

There is no agreed set of diagnostic criteria for DIP. The diagnosis of parkinsonism requires the presence of at least two of four cardinal signs, namely: resting tremor, bradykinesia, rigidity, and impairment of postural reflexes [13,67]. DIP is further defined by a history of symptom-onset after commencing APs or other dopamine-depleting drugs; by an absence of symptoms prior to treatment with these drugs; and by the resolution of symptoms within 6 months of drug withdrawal [14,33].

The most widely used definition of parkinsonism is that of the United Kingdom Parkinson’s Disease Society Brain Bank (UKPDSBB) Clinical Diagnostic Criteria [67]. In their Step 1, they establish that for the diagnosis of a parkinsonian syndrome, bradykinesia must be present, as well as at least one of the following signs: muscular rigidity, a 4–6 Hz resting tremor, or postural instability not caused by primary visual, vestibular, cerebellar, or proprioceptive dysfunction. Step 2 provides exclusion criteria for IPD, including neuroleptic treatment at time of onset, and Step 3 provides prospective supportive positive criteria for IPD.

The Diagnostic and Statistical Manual of Mental Disorders, fifth edition (DSM-V) [68] defines DIP as the presence of resting tremor, muscular rigidity, akinesia, or bradykinesia, developing within a few weeks of starting or raising the dosage of a medication (typically an AP or another neuroleptic), or else after reducing the dose of an antiparkinsonian agent. The presence of bradykinesia is not mandatory, in contrast to the UKPDSBB Step 1 Clinical Diagnostic Criteria [67,69]. 

The Simpson-Angus Scale (SAS) [70] does not consider bradykinesia as an essential sign for diagnosis, but gives a higher importance to rigidity [69]. This scale has been shown to have good internal consistency reliability, and good inter-rater reliability in the assessment of DIP in older patients [71].

#### 3.3.1. Motor Signs

Motor features of DIP have been reported to be similar to those of IPD [7,8,51,72,73,74,75], presenting in a more symmetrical way, with an upper limb predominance [76], and with less axial impairment [6] compared to IPD. In keeping with this, a PET study of DaT in AIP [77] showed that symmetrical parkinsonism was more frequent in patients with normal uptake in the striatal DaTs.

However, there have been reports of cases of DIP with an asymmetrical presentation [72,78] although it is not clear whether these patients could have emerging IPD. It must be noted that the ascertainment of bradykinesia might be difficult in patients with schizophrenia and AIP who have significant negative symptoms [47].

Other studies have reported a greater frequency of concomitant orofacial dyskinesias and of akathisia in cases of DIP compared with IPD [7,8]; absence of resting tremor [8,72]; yet presence of postural tremor [47]. Rabbit syndrome (perioral tremor) is more often associated with AIP [47]. Conversely, amimia has been suggested to be more typical of IPD as well as postural instability [6]. A PET study of DaTs in patients with DIP showed that symmetrical parkinsonism and presence of orofacial dyskinesias were more frequent in patients with normal striatal tracer binding [79].

#### 3.3.2. Non-Motor Signs 

Given the similarities in the motor manifestations of DIP and IPD, it has been thought that non-motor signs might be worth studying to ascertain whether they can assist in distinguishing them. AIP and IPD can cause non-motor signs and symptoms in older individuals, including sleep disturbances, mood changes, autonomic dysfunction, pain, and cognitive deficits [4,80]. Older adults may also have an increased sensitivity to the anticholinergic effects of APs leading to constipation, blurred vision, dry mouth, and urinary retention [81]. Urinary symptoms (urgency, frequency, and nocturia), sleep disturbances (daytime sleepiness, restless legs), concentration problems, sexual dysfunction, and olfactory dysfunction, have all been significantly associated with IPD compared to DIP [4,6,69,80,82]. Olfactory impairment, in particular, is very prevalent in IPD [6,82] and represents an early sign of neurodegeneration; but it is not a common symptom of DIP [69], although this has been reported [83]. It has been suggested that olfactory function tests may be a useful tool in the differential diagnosis between DIP and IPD [84], and also in the diagnosis of “masked” IPD in patients with DIP [6] who do not seem to recover after withdrawal of the offending drug. A study combining olfactory function tests and PET scanning of DaTs, found that patients with DIP and abnormal uptake in the putamen also had an abnormal olfactory function, whilst DIP patients with normal putaminal uptake showed olfactory function similar to control subjects [85]. Another study showed that patients with DIP and normal olfactory test scores had normal cardiac iodine-123-meta-iodobenzylguanidine (MIBG) uptake, and one patient with reduced olfactory test scores showed reduced cardiac MIBG uptake, suggesting that it might be a case of subclinical IPD [84]. Poorer cognitive functioning has been reported in DIP compared to IPD [84]; whilst in patients with schizophrenia, cognitive disturbance was found to be more common in those with AIP than in those without it [80]. 

Olfactory dysfunction and REM Sleep Behaviour Disorder are indicators of early neurodegeneration and hence, of pre-motor IPD. Their presence might be a useful predictor of outcome in DIP, in the sense that they might indicate a group of patients at high risk of developing a synucleinopathy such as IPD [6].

### 3.4. Investigations

#### 3.4.1. Dopamine Transporter Scanning

Single-photon emission CT (SPECT) or PET, using Iodine-123-Ioflupane as a DaT ligand is a well-established sensitive method to help diagnose IPD and other related synucleinopathies, as it can clearly demonstrate loss of presynaptic DaTs in the striatum (particularly in the putamen) in patients with IPD, reflecting the underlying nigrostriatal pathway degeneration [86]. SPECT DaT radioligands available are Iodine-123-beta-CIT, Iodine-123-IPT, and 99mCT-TRODAT-1. However, SPECT using Iodine-123-Ioflupane (DaTscan) is the only imaging technique approved by the European Medicines Agency in 2000, and the US Food and Drug Administration in 2011 to differentiate essential tremor and diseases related to PD [86,87,88]. Since DIP is considered not to be related to nigrostriatal pathway degeneration, this modality of brain scanning has been used to distinguish cases of DIP (normal DaT striatal uptake of tracer) from IPD (reduced striatal uptake) [7,73,75,77,79,82,85,87,88,89,90,91,92,93,94,95,96,97]. Even in the preliminary stages of IPD, DaT uptake in the striatum can be significantly decreased leading to an abnormal DaT scan [98]. By contrast, DIP is considered a form of postsynaptic parkinsonism via D2R blockade and has negligible affinity for DaT, which leads to a normal DaT scan in pure DIP [82,88]. A meta-analysis reported that pooled accuracy measures to differentiate IPD from vascular parkinsonism and DIP were relatively high, with a sensitivity of 85% and a specificity of 80% [87].

A DaT scan can also differentiate between true DIP and individuals with a subclinical form of IPD whose motor symptoms have been unmasked by APs [7,73,80,88,98]. Motor symptoms in IPD do not become manifest until 60–80% of dopaminergic neurons degenerate, so an asymmetrical decrease of DaT uptake in the striatum suggests that these patients may have instead IPD or a subclinical form of the disease [98]. This might be particularly important in cases of DIP that persist over time despite discontinuation of the offending drug.

Certain drugs can interfere with DaT binding and make DaT scans difficult to interpret. This is especially noticeable with dopaminergic stimulants (cocaine, amphetamine-related compounds, methylphenidate, and modafinil) and depleters, which should be stopped prior to scan [88]. Certain aminergic antidepressants, such as sertraline, citalopram, imipramine, and duloxetine can also interfere with DaT binding, but to a much lesser degree [88].

An alternative imaging method to DaT scan is the study of the vesicular monoamine transporter type 2 (VMAT2), using PET and the radioligand [11C](+)-dihydrotetrabenazine which binds to this transporter molecule. This imaging modality has been suggested as an alternative to DaT scan, as the VMAT2 is less modulated by drugs that affect dopaminergic transmission in the brain [51]. In a study using PET to measure binding to VMAT2 in patients with schizophrenia with chronic AIP, patients with schizophrenia without AIP, normal controls, and patients with IPD, observed that in the group with AIP there was a dichotomous striatal binding to VMAT2, with a spared binding subgroup, and a low binding one [51]. In the subgroup with low binding, this reduction was found to be asymmetrical but without the gradient of maximal involvement of the posterior putamen which is typical of IPD [51]. This led the authors to suggest that in a fraction of patients with chronic AIP, VMAT2 binding differs from that of IPD, and might indicate a drug-induced axonopathy that results in synaptic dysfunction [51]. Further, anosmia was found to be the only non-motor parameter that matched this abnormal striatal binding to VMAT2 [51].

#### 3.4.2. Cardiac 123I-MIBG (Iodine-123-Meta-Iodobenzylguanidine) Scintigraphy

Cardiac MIBG scintigraphy is not widely used or funded as a test for IPD in most countries, which may be the reason for the scarcity of studies using this diagnostic technique [82]. Nevertheless, it has been proposed to help diagnose IPD by means of looking at cardiac postganglionic autonomic involvement [99]. Cardiac uptake of the synthetic norepinephrine analog 123I-MIBG depends on the integrity of postganglionic sympathetic neurons, and since α-synuclein-dependent neurodegeneration in IPD affects both pre- and postganglionic autonomic neurons, cardiac 123I-MIBG uptake is impaired [99].

Cardiac MIBG uptake is reported to be significantly reduced in patients with IPD due to sympathetic dysfunction, whilst it is typically normal in those with DIP. This suggests that there is an intact sympathetic function in DIP [100]. A study in patients with DIP, IPD, and control subjects, reported that DIP patients had normal cardiac MIBG scintigraphy compared to those with IPD [100]. In the same study, two patients with DIP and reduced MIBG uptake, showed persistent parkinsonism which responded to levodopa [100].

Patients with DIP and normal olfactory tests have normal cardiac MIBG scintigraphy [84]. It has also been reported that patients with DIP who also have olfactory problems at baseline have altered cardiac MIBG scintigraphy [84,101], indicating that this subset of DIP patients may in fact be suffering from preclinical IPD. 

#### 3.4.3. Substantia Nigra Ultrasonography

Transcranial ultrasonography (TCS) has been considered for its potential in differentiating DIP from IPD. It is a non-invasive, inexpensive, relatively accessible, and radiation free form of investigation. TCS has been widely used to diagnose IPD and also to differentiate Parkinson’s types [40,102]. A study investigated whether substantia nigra (SN) and lenticular nucleus (LN) echogenicity through TCS could differentiate between DIP and IPD [91]. Although their results did not show significant differences, the authors still believed it to be a valid technique, and attributed the lack of significant results to a small sample size. Another study used TCS to measure SN echogenicity and hypothesized that it might be useful in detecting patients with underlying dopaminergic degeneration in those with DIP and at higher risk of not improving following withdrawal of the parkinsonism inducing drug [103]. Results showed that it would be a useful prognostic marker in overall clinical assessment for the estimation of recovery rate in the setting of suspected DIP. However, the paper acknowledged the limitations of this imaging modality compared to DaT radiotracers, and suggested that TCS should not be used to establish a definite diagnosis of the underlying pathology [103]. 

TCS was used in other studies to measure SN echogenicity in IPD, DIP and control groups [40,104]. Results showed that TCS SN echogenicity was significantly increased in IPD, while DIP [40,104] and controls [40] had similar SN echogenicity. The authors concluded that SN echogenicity could be a useful instrument to differentiate IPD from DIP in clinical situations using TCS [40]. However, this study used gastroprokinetic-drugs to induce dopamine blockade, rather than APs.

#### 3.4.4. Others

Magnetic resonance imaging 3-T of the Nigrosome 1 has also been suggested to be helpful in the differential diagnosis of IPD and DIP with high accuracy and may help to screen patients who need DaT imaging in those suspected of having DIP [105].

See Table 5 and Table 6 for the main differences between DIP and IPD in terms of clinical signs and results of investigations, and Table 7 for the relevant investigation studies with their main findings.

## 4. Management of AIP

The management of AIP in older adults generally follows much of the same principles as in working age adults, and is similar to that of other drugs that can cause parkinsonism. There appears to be a significant lack of randomised controlled trials (RCTs) looking at the management of AIP in adults across all age groups, and thus there is a lower level of evidence base when it comes to recommended treatment options. Prior to considering the treatment of AIP, it would be important to remember that prevention is better than cure [69,106]; in particular, by evaluating the risk vs. benefit of antipsychotic medication prior to prescribing [80], and ensuring that the lowest effective dose is used whilst monitoring closely for signs of parkinsonism in high risk groups such as the elderly [7,106]. If an older patient develops symptoms of AIP, it would be prudent to explore whether these symptoms affect their quality of life and activities of daily living; only then should AIP be treated [8,80]. Often, milder cases of AIP do not warrant immediate intervention. The following interventions have been suggested:

### 4.1. Reduce the Antipsychotic Dose

If an intervention is required, the most appropriate first line approach is to reduce the administration of the AP to the lowest effective dose [5,88,107]. One study in patients with very late onset schizophrenia-like psychosis has shown that whilst low dose (100 mg/day) amisulpride could cause a moderate increase in EPS, this was not frequent or severe enough to affect compliance or the benefits of treatment in these patients [108]. This would suggest that in older adults, an initial lower AP dose would be more suitable. This has been confirmed in another recent study of safer prescribing of risperidone for psychosis in Alzheimer’s disease [109]. This study argues for age and Mini-Mental State Examination (MMSE) related dose reductions to avoid treatment emergent EPS (including parkinsonism), with a pragmatic approach of “starting low and going slow”, whilst preserving clinical efficacy [109]. Reduction of antipsychotic dose is often neglected, with one study showing that in a third of older patients prescribed anticholinergic medication for AIP there had been no attempt to reduce their AP dose [110].

### 4.2. Stop the Antipsychotic

Wherever possible, it would be recommended to stop the offending drug [69,88,111]. Because APs have long half-lives, the improvement in AIP will not be immediate [107]. Unfortunately, stopping or reducing the dose of APs can cause a relapse in psychotic symptoms in many patients, posing greater risks to themselves and others.

### 4.3. Switch Antipsychotic

In order to reduce the risk of relapse from stopping the offending AP, it would be prudent to consider switching to an alternative AP [47,98]. SGAs have a reduced propensity to cause parkinsonism at lower doses, with quetiapine and clozapine being deemed the APs of choice [2,69,88]. Although evidence has shown that there is little difference in the risk of relapse with immediate versus gradual stopping or switching of APs, most psychiatrists would use a cross-titration strategy. When switching APs, the preferred strategy is usually to cross-titrate medication whereby there is a reduction of the first AP whilst introducing and increasing the second AP [112].

Quetiapine is widely used for psychosis in patients with IPD [82]. Clozapine has the least potential to cause parkinsonism, and is also frequently used to treat psychosis in IPD where it significantly improves psychosis without worsening motor symptoms [113]. The reduced parkinsonian liability of these newer agents derives from their propensity to impose more 5-HT receptor blockade, and in some cases less D2R blockade relative to conventional APs [5]. Unfortunately, due to its significant side-effects, clozapine is often not appropriate to manage psychosis in older patients. Side-effects include agranulocytosis, orthostatic hypotension, tachycardia, myocarditis, metabolic syndrome, and diabetes. Furthermore, older patients are more sensitive to the anticholinergic side-effects of clozapine such as confusion, constipation, and urinary retention [114]. 

### 4.4. Add Anticholinergic

Adding anticholinergic medication to help with AIP is frequently seen in clinical practice and is supported by anecdotal evidence and small studies [81,88]. However, their true efficacy remains uncertain, and is potentially overestimated [72,115]. Anticholinergics are available in oral and parenteral forms [107]. There is little difference between the efficacy of the two most popular anticholinergics: benztropine and trihexyphenidyl [7]. Unfortunately, anticholinergic medication can cause numerous side-effects including urinary retention, angle closure glaucoma, exacerbation of cognitive impairment, worsening tardive dyskinesia, tachycardia, constipation, and increased risk of delirium [47,81,82,107]. Such side-effects are more common when prescribed with other medication with potential for anticholinergic adverse effects [116]. Furthermore, anticholinergics may reduce the clinical efficacy of AP agents [107]. Given the significant side-effect burden, and the fact that older patients are more susceptible to them, it is recommended to avoid anticholinergic medication to treat AIP in this group [2]. If prescribed, treatment should only be continued if there are signs of clear improvement in the parkinsonism [7], with a typical duration of 3 months [116]. Treatment should be regularly reviewed in order to assess the need for continued use, and it should be eventually stopped. Prophylactic use of anticholinergics is not recommended particularly in older patients [107].

### 4.5. Add Amantadine

Amantadine has antidyskinetic effects and is often used in the treatment of IPD. Its mechanism of action is not fully understood, but it may enhance dopamine release and inhibit its re-uptake into the presynaptic nerve terminal in the striatum [117]. Amantadine has been shown to be beneficial in treating AIP in small clinical trials [8] with some studies finding it to have similar efficacy to anticholinergic medication [118]. However, some of the efficacy data is weak and conflicting [115], with one study showing no difference compared to placebo [119]. It is clear that further robust clinical trials would be required in order to fully ascertain the efficacy of amantadine on older patients with AIP. Amantadine is better tolerated by older patients [2,116] and has been shown to cause fewer side-effects than anticholinergic medication [118]. However, it still can cause some adverse effects including dizziness, insomnia, nausea, dry mouth, blurred vision, and at high doses, it can exacerbate psychosis [116]. Since the drug is excreted primarily in the urine, a lower dosage is required in older people [107]. 

### 4.6. Add L-Dopa or Dopamine Agonist

For those with AIP who do not have underlying IPD the concurrent use of AP drugs with dopaminergic drugs is generally contraindicated. Levodopa and D2R agonists such as bromocriptine are usually ineffective in treating AIP (due to striatal D2 receptor blockade), and can exacerbate psychosis [7,88,107]. Drugs with direct dopaminergic agonist effects such as lisuroride or pergolide can cause confusion in older people [120].

### 4.7. Electroconvulsive Therapy 

Beneficial effects of electroconvulsive therapy (ECT) on both psychiatric complications and motor symptoms of IPD have been observed since 1947 [121]. In refractory cases of AIP, where previous management steps have not been successful in ameliorating parkinsonism, case reports suggest that ECT could be an effective option for older patients [122,123], although the evidence base is weak, with no RCTs available. A proposed mechanism of the antiparkinsonian effect of ECT is that it increases the sensitivity of dopamine receptors [121].

### 4.8. Physiotherapy

Physiotherapy may also prove beneficial to patients with AIP, particularly those with disordered posture and gait [69].

## 5. Prognosis of AIP

AIP is generally considered an acute condition having its onset within the first week of starting the antipsychotic [5]. Despite continuation of the offending medication, parkinsonism may gradually abate (and this is suggested to be due to dopamine receptor blockade gradually decreasing over time) [1]. If the offending medication is stopped, AIP gradually disappears in the significant majority of cases, although this can take up to 18 months. This time delay has been related to the possible persistence of some AP drugs in tissue after drug withdrawal [1]. Estimates for the persistence of parkinsonism appear to vary. Most studies focus on DIP more generally rather than specifically to AIP. Estimates on persistence of AIP following discontinuation of APs vary from as low as 1% [1] up to 20% [8]. If symptoms persist, it is worth considering whether the patient may have had subclinical IPD before starting treatment. Some have also argued that persistent parkinsonism after drug withdrawal may be due to a direct toxic effect on the dopaminergic system [106]. Early differentiation of IPD from AIP is crucial as it has vastly different prognostic implications [111]: receptor imaging studies have shown that over a third of patients with DIP have nigrostriatal dopaminergic dysfunction [73]; and structural imaging studies revealed that microstructural white matter changes are present over various brain regions in patients with DIP [65]. A study measuring functional connectivity (FC) in DIP showed that FC between the sensorimotor network and the cerebellum or prefrontal region was lower in the group of DIP patients with partial recovery following drug withdrawal compared to the complete recovery group; hence, it was a predictor of whether patients with DIP would recover within 3 months of withdrawal of the offending drug [66].

## 6. Conclusions

DIP is a common problem encountered in clinical practice, and is considered to be the second most frequent cause of parkinsonism after IPD, especially in the older population, ranging from 2% to 76% of all cases of parkinsonism. APs are one of the most implicated drug groups, with estimations suggesting that over 50% of subjects over 60 years of age on long-term therapy with APs develop AIP. Low-potency FGAs are significantly less likely to cause AIP than SGAs and high-potency FGAs, and the risk of AIP with high-dose SGAs does not seem to differ substantially from that resulting from FGAs. DIP caused by D2R antagonists such as APs and gastrokinetic drugs seems to appear within 6 months of treatment initiation. The most important risk factors are age over 60 and female gender, whilst other risk factors include organic brain damage, learning disability, dementia, IPD, hypertension, organic personality disorder, and diagnoses of schizophrenia, mania, depression or anxiety, HIV infection, non-European ancestry, and presence of HLA-B44 in addition to use of high potency APs, higher dosage of APs, and long-term exposure to APs. The diagnosis of DIP could triple the lifetime risk of developing IPD. Pathophysiological understanding of DIP is incomplete. The main but not sufficient mechanism is the blockade of D2Rs in the striatum, but it has also been proposed that the rate APs dissociate from the D2R may predict their likelihood to cause parkinsonism; with transient D2 blockade (fast dissociation) being less likely (e.g., clozapine, quetiapine), in addition to gene expression changes induced by APs. D2R antagonists may also have a direct neurotoxic effect on dopaminergic neurons. This may also explain why some patients continue to experience parkinsonism many months following discontinuation of the offending drug (around 20% of patients). Motor features of AIP are similar to those of IPD; more symmetrical and with an upper limb predominance and with less axial impairment compared to IPD. Concomitant orofacial dyskinesias, akathisia, perioral tremor, and absence of resting tremor are more frequent in AIP than IPD. The most helpful symptoms to differentiate AIP from IPD are olfactory deficits and REM sleep behaviour disorder, as these are invariably more related to neurodegeneration than to side-effects from drugs. The most widely used and diagnostically useful investigation is SPECT/PET imaging of the DaTs. Decreased binding to DaT is a well-established biomarker of IPD and other synucleinopathies, and it is not expected to be found in AIP. Patients with suspected AIP and abnormal DaT scans, especially with asymmetric findings, most likely suffer from IPD. There is a lack of RCTs on the pharmacological management of AIP in all age groups, hence there is a lower level of evidence base to recommend treatment options. The most judicious approach is prevention of AIP, considering the pros and cons of initiating APs, especially in those in higher risk groups. If an AP is to be commenced, a SGA at low dose should be used, and high-potency FGAs should be avoided. If AIP occurs, the first step should be a dose reduction, followed by discontinuation or switching to an alternative AP if possible. For the specific treatment of AIP in older adults, there is little evidence for effective pharmacological interventions. More research is required into the pathophysiological mechanisms underpinning AIP beyond the blockade of D2Rs, such as gene expression changes, which should lead to personalised prescribing, and into the pharmacological management of AIP using an RCT methodology. Clinicians should also consider more closely the patient and drug-related risks when prescribing APs. 

## Figures and Tables

**Table 1:** Prevalence and Incidence of DIP.

Study *	**Prevalence of DIP**
Estevez-Fraga et al., 2018 Review article	Globally over 50% of aged 65 and over
Hoffman et al., 1987 Cross-sectional study in inpatients and outpatients (*n* = 21)	76%
Morgante et al., 1996 Prevalence survey in the community population study (*n* = 24,496)	32.7/100,000
Benito-Leon et al., 2003 Epidemiological study in the community (*n* = 5278)	0.5%
Barbosa et al., 2006 Community based survey (*n* = 1186)	2.7%
Fleury et al., 2008 Cross-sectional prevalence study in the community (*n* = 2312)	21.7/100,000
Buyn et al., 2019 Korean National Health Insurance Claims Database (*n* = 1285)	4.09/100,000 in 2009 and 7.02/100,000 in 2015
Han et al., 2019 Korean National Health Insurance Review and Assessment Service Database (*n* = circa 50 million)	9.78/100,000
Khedr et al., 2015 Cross-sectional community based survey (*n* = 8027)	37/100,000
Tse et al., 2008 Cross-sectional study in nursing homes *n* = 28/397 had parkinsonism, 2 of which had DIP	0.5%
Moghal et al., 1995 Survey in nursing homes (*n* = 67)	3%
Study *	**Incidence of DIP**
Caligiuri et al., 1999 Longitudinal prospective study in psychiatric outpatients (*n* = 120)	28.6%
Rajpur et al., 1984 Epidemiological study in the community (*n* = 138 new cases of parkinsonism)	7.2% (of 138)
Bower et al., 1999 Epidemiological study in the community (*n* = 364 incident cases of parkinsonism)	20% (of 364)
Baldereschi et al., 2000 Longitudinal study in the community (*n* = 3084 of which *n* = 68 had parkinsonism)	10% (of 68 cases)
Rocca et al., 2001 Epidemiological study in the community (*n* = 2739 of which *n* = 364 with parkinsonism)	20% (of 364)
Benito-Leon et al., 2004 Epidemiological study in community (*n* = 3813, of which *n* = 68 parkinsonism)	32.3% (of 68)
De Lau et al., 2004 Prospective community population cohort (*n* = 6839)	12%
Munhoz et al., 2010 Cohort study in outpatient service (*n* = 1528)	7.9%
Seijo-Martinez et al., 2011 Community-based survey (*n* = 41)	31.7%
Bondon-Guitton et al., 2011 French Pharmacovigilance Database (*n* = 20,855)	0.7%
Savica et al., 2013 Cohort Study in community population (*n* = 542)	6.6%
Savica et al., 2017 Epidemiological study in community population (*n* = 906)	11.9%
Vale et al., 2018 Cross-sectional study in community population (*n* = 610)	12.3%
Druschky et al., 2020 German Pharmacovigilance Database (*n* = 340,099)	0.08%
De Germay et al., 2020 WHO Pharmacovigilance Database (*n* = 9,009,107)	0.05%
Han et al., 2019 Korean Health Insurance Review and Assessment Service Database (*n* = circa 50 million)	8.69/100,000
Fleury et al., 2018 Retrospective Incidence study in community population (*n* = 2312)	2.5/100,000

* 3, 8, 9, 10, 11, 12, 13, 14, 15, 16, 17, 18, 19, 20, 21, 22, 24, 25, 26, 27, 28, 29, 32, 35, 36.

**Table 2:** Groups of antipsychotic and other anti-D2R drugs.

	First Generation APs	Second Generation APs
Phenothiazines	Chlorpromazine Promazine Levomepromazine Triflupromazine Mesoridazine Thioridazine Fluphenazine (HP) Perphenazine Prochlorperazine (HP) Trifluoperazine (HP)	
Butyrophenones (HP)	Haloperidol Benperidol Droperidol	
Thioxanthenes	Chlorprothixene Clopenthixol (HP) Flupenthixol (HP) Thiothixene Zuclopenthixol (HP)	
Benzamides	Sulpiride Tiapride Veralipride Levosulpiride Metoclopramide Mosapride Lisepride Clebopride	Amisulpiride (FD) Remoxipride (FD) Sultopride Itopride
Indole derivatives	Oxypertine Molindone	Ziprasidone Lurasidone
Diphenylbutylpiperidines	Pimozide (HP)	
Other		Loxapine Clozapine (FD) Olanzapine Quetiapine (FD) Asenapide Clotiapine Zotepine Paliperidone Sertindole Aripiprazole

D2R = Dopamine 2 receptor; AP = Antipsychotic; HP = High Potency, FD = Fast Dissociation.

**Table 3:** Studies highlighting specific drugs associated with Drug-induced Parkinsonism and their frequencies.

Study (3, 20, 26, 27, 38, 40, 41)	Drug Class	% and Number of Subjects
Munhoz et al., 2010	Antipsychotics	52.9% *n* = 74
	Calcium Channel Blockers	35.7% *n* = 50
	Other drug classes	11.4% *n* = 16
Bondon-Guitton et al., 2011	Central dopaminergic antagonists	49% *n* = 128
	Antidepressants	8% *n* = 21
	Calcium Channel Blockers	5% *n* = 13
	Peripheral dopaminergic antagonists	4.6% *n* = 12
	H1 antihistamines	4.6% *n* = 12
	Miscellaneous drugs	28.7% *n* = 75
Druschky et al., 2020	Antipsychotic Drugs:	
	-First Generation—Low Potency	0.024% *n* = 17
	-First Generation—High Potency	0.159% *n* = 78
	-Second Generation	0.073% *n* = 139
Munhoz et al., 2017	Classic neuroleptics	*n* = 78
	-Haloperidol	48.7% *n* = 38
	-Levomepromazine	24.4% *n* = 19
	-Chlorpromazine	17.9% *n* = 14
	-Thioridazine	9% *n* = 7
	Second-generation neuroleptics	*n* = 21
	-Risperidone	81% *n* = 17
	-Olanzapine	19% *n* = 4
	Calcium channel blockers	*n* = 58
	-Flunarizine	65.5% *n* = 38
	-Cinnarizine	34.5% *n* = 20
De Germay et al., 2020	Risperidone	14% *n* = 637
	Haloperidol	9.4% *n* = 428
	Aripiprazole	7.2% *n* = 330
	Olanzapine	6.2% *n* = 283
	Valproic acid	5.7% *n* = 262
	Quetiapine	4.0% *n* = 184
	Sulpiride	3.6% *n* = 164
	Clozapine	3.5% *n* = 160
	Metoclopramide	3.5% *n* = 160
	Paliperidone	3.3% *n* = 151
Oh et al., 2018	Levosulpiride	78.2% *n* = 54
	Metoclopramide	11.58% *n* = 8
	Clebopride	7.24% *n* = 5
	Itopride	2.89% *n* = 2
Kim S et al., 2019	Typical Antipsychotics	0.3% *n* = 15
	Atypical Antipsychotics	0.8% *n* = 45
	Gastrokinetic	22.2% *n* = 1222

**Table 4:** Risk factors for AIP.

Patient-Related	Drug-Related
Age > 60	High potency first generation antipsychotics
Female Gender	High dose of antipsychotics (first and second generation)
Organic Brain Damage	Long-term exposure to antipsychotics
Intellectual Disability	
Dementia	
Idiopathic Parkinson’s Disease	
Hypertension	
Non-European ancestry	
HIV infection	
HLA-B44	
Schizophrenia, depression	

AIP = Antipsychotic-Induced Parkinsonism; HIV = Human Immunodeficiency Virus; HLA = Human Leukocyte Antigen.

**Table 5:** Comparison of DIP vs. IPD symptomatology and investigation findings.

	AIP	IPD
**General**		
Symptoms	More symmetrical	More asymmetrical
Onset	Acute or subacute	Chronic
Course	Reversible after withdrawal of drug (*)	Progressive
**Motor**		
Upper Limb Predominance	↑	↑↓
Axial Impairment	↓	↑
Oro-facial dyskinesias	↑	↓
Akathisia	↑	↓
Resting Tremor	↓	↑↑
Postural Tremor	↑	↓
Perioral Tremor	↑	↓
Amimia	↓	↑
Postural instability	↓	↑
**Non-motor**		
Mood Changes	↑	↑
Autonomic Dysfunction	↑	↑↑
Cognitive Deficits	↑	↑↑
Pain	↑	↑
Sleep disturbances	↑	↑↑↑
Olfactory dysfunction	↓↓↓	↑↑↑
Urinary symptoms	↑	↑↑
Concentration Problems	↑	↑↑↑
Sexual dysfunction	↑	↑↑

↑ = Increased frequency, ↓ = Decreased frequency; AIP = Antipsychotic-Induced Parkinsonism; IPD = Idiopathic Parkinson’s Disease; * Unless associated neurodegeneration of striato-nigral pathway is present.

**Table 6:** Investigation findings in DIP vs. IPD.

Investigation	AIP	IPD
DaT Scan	Normal (*)	Abnormal/Frequently asymmetrical findings
Cardiac MIBG Scintigraphy	Normal (*)	Abnormal
TCS of Substantia Nigra	Normal (*)	Abnormal

AIP = Antipsychotic-Induced Parkinsonism; IPD = Idiopathic Parkinson’s Disease; DaT = Dopamine Transporter; MIBG = meta-iodobenzylguanidine; TCS = Transcranial Ultrasonography. * Unless associated neurodegeneration of striato-nigral pathway is present.

**Table 7:** Studies on investigations for the differential diagnosis of DIP vs. IPD.

Study *	N	Conclusions
**DAT** **Scanning**		
Lorberboym et al., 2006	30	[123I]FP-CIT SPECT can help distinguish whether DIP is drug-induced or an exacerbation of subclinical IPD.
Diaz-Corrales 2010	79	DIP and IPD are clinically difficult to differentiate, and can be improved by [123I]FP-CIT SPECT imaging.
Shin et al., 2015	92	Symmetrical parkinsonism was more prevalent and duration of drug exposure before the onset of parkinsonism shorter for patients with normal vs. abnormal [18F]FP-CIT PET scans.
Tinazzi et al., 2009	19	[123I]FP-CIT SPECT imaging helps identify subjects with DIP secondary to a loss of dopamine nerve terminals in the context of a progressive degenerative parkinsonism.
Bovi et al., 2010	48	Patients with DIP and pathological putamen uptake had abnormal olfactory function. Smell deficits in DIP patients may be more associated with dopaminergic loss than drug-mediated dopamine receptor blockade.
Tinazzi et al., 2012	97	D2-receptor blockade may accompany a dopamine nigrostriatal terminal defect, as assessed by [123I]FP-CIT SPECT abnormalities, in an applicable proportion of DIP patients.
Jin et al., 2013	98	Dual-phase [18F]FP-CIT PET imaging helps demonstrate striatal DAT loss in neurodegenerative parkinsonism.
Park et al., 2014	33	[18F]FP-CIT PET imaging useful to differentiate parkinsonism in patients with inconclusive parkinsonian features, except in patients who show atypical features or who eventually progress to PD.
Sadasivan et al., 2015	65	[18F]FP-CIT PET can significantly impact patient clinical management in those with clinically uncertain parkinsonian syndromes in a tertiary referral center.
Bega et al., 2015	83	[18F]FP-CIT PET had a significant impact on clinical diagnosis and management.
Hong et al., 2016	50	Persistent DIP in patients with visually normal [18F]FP-CIT PET DAT imaging may be associated with subtle reduction of DAT activity.
Bhattacharjee et al., 2017	48	Compliance of the [123I]FP-CIT SPECT imaging with the existing standard guidelines is good and influences the clinical diagnosis and management in 23% of the patients with parkinsonism.
Vlaar et al., 2008	248	[123I]FP-CIT SPECT is accurate to differentiate patients with IPD from those with essential tremor (ET), and IPD from vascular parkinsonism (VP) and DIP.
**VMAT using PET and radioligand**		
Galoppin et al., 2020	45	Striatal VMAT2 binding is abnormal in a fraction of chronic DIP cases and differs in spatial distribution from PD.
**Cardiac Scintigraphy**		
Lee et al., 2006	20	MIBG uptake was not different between the DIP patients and controls. Two DIP patients whose MIBG uptake was significantly reduced showed persistent parkinsonism and responded dramatically to levodopa.
Lee et al., 2007	15	An olfactory function test may be useful to detect DIP unrelated to PD and to identify patients with DIP who have subclinical PD.
**Transcranial Ultrasonography of the Substantia Nigra**		
Oh et al., 2018	193	SN echogenicity on TCS could help differentiate PD from DIP in clinical situations. Pure DIP and unmasked PD exhibited different SN echogenicity patterns. Early SN echogenicity findings on TCS could be used as a biomarker to predict clinical prognosis of DIP.
López-Sendón Moreno et al., 2016	60	SN hyperechogenicity assessed with TCS is a valid prognostic marker in the setting of suspected DIP.

N = number of subjects; DIP = Drug-induced Parkinsonism; ET = Essential Tremor; FP-CIT = N-3-fluoropropyl-2beta-carbomethoxy-3beta-4-iodophenyl Tropane; F18 = Fluorine-18; IPD = Idiopathic Parkinson’s disease; MIBG = 123I-metaiodobenzylguanidine; PET = Positron Emission Tomography; SN = Substantia Nigra; SPECT = Single-Photon Emission Computerized Tomography; TCS = Transcranial Ultrasonography; VMAT2 = Vesicular Monoamine Transporter 2; VP = Vascular Parkinsonism; 123I = Iodine-123. * 40, 51, 73, 75, 77, 79, 84, 85, 90, 91, 92, 93, 94, 95, 96, 97, 100, 102, 103.

## Data Availability

Not applicable.
